# The Influence of Various Smoking Categories on The Risk of Gestational Hypertension and Pre-Eclampsia

**DOI:** 10.3390/jcm9061743

**Published:** 2020-06-04

**Authors:** Małgorzata Lewandowska, Barbara Więckowska

**Affiliations:** 1 Medical Faculty, Lazarski University, 02-662 Warsaw, Poland; 2 Division of Gynecological Surgery, University Hospital, 33 Polna Str., 60-535 Poznan, Poland; 3 Department of Computer Science and Statistics, Poznan University of Medical Sciences, 60-806 Poznan, Poland; barbara.wieckowska@ump.edu.pl

**Keywords:** smoking, smoking cessation, gestational hypertension, pre-eclampsia, pregnancy, underweight

## Abstract

The relationship between smoking and the risk of pregnancy-induced hypertension (PIH) is not clearly established. Therefore, we conducted an analysis of cigarette smoking in a Polish cohort of women, recruited in the first trimester of a single pregnancy in 2015–2016. We evaluated the women who subsequently developed PIH (*n* = 137) (gestational hypertension—GH (*n* = 113) and pre-eclampsia—PE (*n* = 24)), and the women who remained normotensive (*n* = 775). The diseases odds ratios (and 95% CI—confidence intervals) were calculated in a multivariate logistic regression. In the PIH cases (vs. normotensive women) we found more smokers (25.6% vs. 17.2%, *p* = 0.020) including smokers in the first trimester (14.6% vs. 4.8%, *p* < 0.001). The average number of cigarettes smoked daily per smokers in the first trimester was 11.2 (range 2–30), and the average number of years of smoking was 11.6 (range 2–25). The number of years of smoking was a stronger risk factor for GH and PE than the number of cigarettes/day. Compared to the women who have never smoked, smoking ever before pregnancy was associated with a higher GH risk (AOR = 1.68; *p* = 0.043), and with no effect on PE risk (OR = 0.97; *p* = 0.950). Smokers in the first trimester had a higher odds ratio of GH (AOR = 4.75; *p* < 0.001) and PE (OR = 2.60; *p* = 0.136). Quitting smoking before pregnancy (ex-smokers) was associated with a lower odds ratio of GH (AOR = 0.83; *p* = 0.596) and PE (OR = 0.33; *p* = 0.288). However, quitting smoking during pregnancy was associated with a higher risk of GH (AOR = 11.63; *p* < 0.0001) and PE (OR = 3.57; *p* = 0.238). After dissection of the cohort into pre-pregnancy body–mass index (BMI) categories, smoking in the first trimester was associated with the higher hypertension risk in underweight women (OR = 22.00, *p* = 0.024). Conclusions: The factors that increased the risk of GH and PE were smoking in the first trimester and (paradoxically and more strongly) smoking cessation during pregnancy. Our results suggest that women of childbearing potential should be encouraged to quit smoking before pregnancy.

## 1. Introduction

Pregnancy-induced hypertension (PIH) is characterized by an increase in de novo blood pressure after the 20th week of pregnancy [1]. It affects an average of about 7–10% of pregnant women and includes isolated gestational hypertension (GH) and pre-eclampsia (PE) [1,2,3]. PE is accompanied by organ disorders and it is one of the main causes of morbidity and mortality of mothers and fetuses. GH has a milder course (not associated with organ disorders), but also increases the risk of adverse pregnancy outcomes, compared to normotensive women. There remains doubt as to whether these are separate diseases. However, they have been shown to have many common risk factors as well as pathogenesis elements [1,4]. In the pathomechanism of PE, the primary role of trophoblast invasion disorders in the period of placenta development has been demonstrated, which results in ischemia and hypoxia of the placenta, an increase in oxidative stress and the initiation of numerous processes leading ultimately to endothelial damage [5,6,7]. The result is the increase in blood pressure [1]. Numerous studies have found dyslipidemia, inflammation, oxidative stress as the main element of the pathogenesis of GH and PE [1,2,4,6]. It has been previously shown that lower levels of antioxidants in early pregnancy may increase the risk of PIH [8,9].

There are significant discrepancies in the relationship between smoking and the development of PIH [10,11,12,13]. Establishing these relationships is important because it is estimated that around a quarter of the population in the European Region smokes [14,15], and maternal smoking is the most prevalent preventable cause of pregnancy complications such as neonatal morbidity, low birthweight, and premature delivery [16]. Many studies have shown that smoking during pregnancy (paradoxically) reduces the risk of PE. However, there are also opposite results, and mechanisms confirming the beneficial or harmful effects of smoking on this risk have not been established [11,12,13]. Tobacco smoke contains more than 7000 chemicals, among them various toxic and carcinogenic and cocarcinogenic substances [8,9,11,12,17,18,19,20]. Smoking has been shown to trigger inflammatory processes and to increase oxidative stress, as well as to reduce placental flows [16,17]. On the other hand, the study by Laule et al. indicates that nicotine can have anti-inflammatory effects through the α7-nAChR (acetylcholine receptor subunit) and inhibition of pro-inflammatory cytokine production, which may attenuate hypertension caused by placental ischemia. At the same time, this beneficial effect can be overshadowed by other effects of nicotine on nicotinic receptors and the ability to increase systemic blood pressure (directly) [11]. Another component of cigarette smoke, carbon monoxide (CO), has the property of reducing vascular tone and inhibiting inflammatory cascades [11].

Both systematic reviews by England et al. (covering 48 epidemiologic studies from 1959–2006) and a systematic review by Conde-Agudelo et al. (covering 35 studies from 1966–1998) showed the inverse relationship between smoking during pregnancy, and PE [12,13]. However, several studies have shown that smoking during pregnancy increased the risk of hypertension in pregnancy [10,21,22,23,24]. In a population-based prospective cohort study, Bakker et al. found a slight increase in the risk of PE for smoking in the first trimester [22]. In a prospective study of 508 single-pregnant women in the 16–22th week, Rauchfuss et al. found that women who never smoked and those who reduced the number of cigarettes smoked during pregnancy had the lowest risk of PIH; and the women who quit smoking before or during pregnancy increased the risk of PE [23]. On the contrary, in a registry-based study, Kharkova et al., found negative relationship between smoking and PE risk and the risk was the same in the women who quit smoking during the first trimester [25]. Different designs and methodologies of research, including various risks of the studied populations or various sample size, could have influenced the discrepancy. Studies found in the literature focused mainly on the assessment of PE.

Our study is one of the first to comprehensively assess smoking in Polish pregnant women for the risk of GH and PE separately. In a multivariate statistic model we examined the risk of the diseases for several categories of smoking of cigarettes: smokers (ever before pregnancy), the women who quit smoking before pregnancy (ex-smokers), smokers in the first trimester, those who quit or reduced smoking during pregnancy, or smoked unchanged until the end of pregnancy, as well as considering the length of smoking time, number of cigarettes, and pack-years. In addition, we conducted a study after dissection into pre-pregnancy body–mass index (BMI) categories.

## 2. Experimental Section

The study was conducted in accordance with the Helsinki Declaration. It was approved by the Bioethics Committee of the Medical University of Poznan, Poland (approval number 769/15). All the participants signed the Informed Consent Form. 

### 2.1. Method and Study Population 

The current analysis was conducted in a prospective cohort of pregnant women recruited in the end of the first trimester. The participants were recruited at the Obstetric-gynecological and Neonatological Center (Poznan, Poland); It is a III-grade reference center, with 6000–8000 deliveries a year. The participants were recruited in 2015–2016; Pregnancy outcomes were collected in 2016–2017; Statistical Analyses were conducted in 2017–2019.

We recruited white (Caucasian) women of descent from one region of Poland (Wielkopolska) in the 10 (+0) − 14 (+6) th week of a single pregnancy without aneuploidy. The criteria also included delivery of a phenotypically normal child ≥25th gestational week, and mother’s age at conception between 18–45.

The criteria for excluding candidates from the original cohort were chronic diseases, e.g., hypertension or/and diabetes mellitus, and kidney or liver diseases, as well as immunological and inflammatory diseases, and thromboembolism.

Women’s characteristics were collected using a questionnaire. The participants filled it out themselves (but in the presence of midwives) during recruitment. We collected the information such as age, height, and pre-pregnancy weight. The set of data also included current pregnancy history, obstetrical and gynecological histories, and concurrent diseases, as well as socioeconomic and demographic characteristics (education status, financial status, and place of residence, and marital status). We also collected the information about the use of multivitamins and medications, as well as about family history. Importantly (especially for the current analysis) we collected information about smoking. All women declared consuming no alcohol in pregnancy.

After the end of pregnancy, outcomes were taken from medical records. All pregnancy outcomes (among others, birth weight and intrauterine growth restriction (IUGR), gestational age at birth and preterm birth, as well as fetal sex and APGAR results) and pregnancy complications (e.g., hypertension in pregnancy (PIH) and its main forms, and gestational diabetes mellitus (GDM) and its main forms) have been recorded.

The recruitment was conducted among women taking typical laboratory tests. Information about the study was available to everyone in the laboratory. Participation in the study was voluntary. It was proposed to each of 1300 women who had the adopted criteria, in 2015–2016 (in the period of 12 months). Missing data concerned 388 (29.9%) women (the participants who did not meet the inclusion criteria after the end of pregnancy, as well as women whose data were incomplete). The original cohort consisted of 912 women.

In the current analysis, the study group (*n* = 137) consisted of the women who subsequently developed hypertension in pregnancy (113 women developed GH and 24 women developed PE), and the control group (*n* = 775) consisted of the participants who remained normotensive. The aim of the current analysis was to assess the relationship between several categories of smoking, and the risk of hypertension in pregnancy (GH and PE), in the whole cohort and after breaking down into maternal pre-pregnancy BMI categories.

### 2.2. Studied Variables

Information on cigarette smoking addiction was self-reported. In the current analysis we evaluated the following smoking categories: women who have never smoked, smokers (all smokers before and during pregnancy), ex-smokers (women who quit smoking before pregnancy), smokers in the first trimester, those who quit smoking during pregnancy (in II-III trimester), smokers who reduced smoking during pregnancy (in II-III trimester), those who smoked unchanged until the end of pregnancy, considering the number of years of smoking, the number of cigarettes smoked a day and pack-years. The value of pack-years of smoking for each woman was the result of multiplying the number of years of smoking and the size of a packet of cigarettes (calculated as a ratio of the number of cigarettes smoked per day/20 cigarettes in a packet). We compared smoking categories in the case and control group. 

Height and pre-pregnancy weight were self-reported. The height was also measured in the hospital (before delivery) and the values from hospital records were included in the analysis. Pre-pregnancy BMI (a ratio of the weight and the height^2^, kg/m^2^) was calculated for each woman). Normal BMI was defined as 18.5–24.99 kg/m^2^. Underweight, overweight and obesity were defined as BMI <18.5 kg/m^2^, 25.00–29.99 kg/m^2^ and ≥30.00 kg/m^2^, respectively.

Gestational weight gain (GWG) was calculated as the difference between the weight measured before delivery (data available in the medical records) and the pre-pregnancy weight. The GWG ranges recommended by the US National Academy of Medicine (formerly the Institute of Medicine) were defined as 12.5–18 kg for underweight women, 11.5–16 kg for normal BMI, 7–11.5 kg for overweight women and 5–9 kg for obese women.

### 2.3. Studied Pregnancy Complication

Pregnancy outcomes were taken from the medical records. PIH was defined in accordance with the Polish guidelines (2015), as “arterial pressure equal to and higher than 140/90 mmHg (on two occasions, at least 4 h apart, in a sitting position, with an oscillometric device) developed de novo after the 20th week of pregnancy, receding up to 12 weeks after delivery.” PIH includes two (main) forms. “Gestational hypertension (GH) was diagnosed if no other disturbance was found; PE was diagnosed when any of the following appeared de novo: Proteinuria (≥300 mg/day or ≥0.3 g/L; protein/creatinine ratio ≥0.3; 1+ in the strip test); thrombocytopenia <100 G/L; worsening of renal function; damage to the liver function; pulmonary edema; symptoms from the central nervous system; blurred vision. Intrauterine growth restriction (IUGR) was not one of the criteria of diagnosis”. Only proteinuria was found in all cases of PE in this cohort (≥0.3 g/L).

The mother’s arterial pressure (systolic and diastolic) was measured before and after delivery, and the mother’s arterial pressure from a postpartum ward were included in the analysis. Values of blood pressure before pregnancy were self-reported.

### 2.4. Statistical Analyses

Statistical analyses were performed using the Statistica 13 software. Before statistical analyses, the Shapiro–Wilk test was used to assess the normality of the data distribution. The Mann–Whitney *U* test was applied for comparisons of continuous variables; means and medians were given as description. For binomial categories, the Pearson chi-square test (or Fisher exact test when Cochran assumption was not met) was used (*p*-value < 0.05 was assumed to be significant). Only available data were taken into consideration.

The impact of smoking on the risk of developing PIH and its forms (isolated GH and PE, separately) was assessed in the logistic regression. *p*-value was calculated using the Wald test (*p*-value < 0.05 was considered to be significant). Odds ratios of PIH (and 95% confidence intervals CI) were calculated for smoking categories expressed as continuous variables, and for each smoking category expressed as categorical variables with respect to the reference category which was assigned OR = 1.00; the reference category was “women who have never smoked” or “smokers in the first trimester”.

Crude odds ratios (OR) of PIH for smoking categories were calculated in the univariate logistic regression, and adjusted odds ratios (AOR) of PIH for smoking categories were calculated in the multivariate logistic regression after adjusting for maternal age, primiparous, pre-pregnancy BMI, GWG outside the range of the recommendations regardless of the BMI category, prior PIH, and infertility treatment.

Graphs of PIH risk were made based on a one-and multidimensional logistic model regression models presenting the odds ratios (OR and AOR) and 95% confidence intervals on a logarithmic scale (using the PQStat software).

## 3. Results

Characteristics of participants in the control and study groups are presented in Table 1 and Table S1.

In the whole cohort (Table S1), 775 (85%) women remained normotensive and 137 (15%) women developed PIH, including GH (*n* = 113) and PE (*n* = 24). In the cohort, 168 (18.4%) women were smokers, 111 (12.1%) women quit smoking before pregnancy, and 57 (6.3%) smoked in the first trimester (22 women quit smoking during pregnancy, 10 women reduced smoking during pregnancy, and 25 women smoked unchanged until the end of pregnancy). The average number of cigarettes smoked daily per person among the smokers in the first trimester was 11.2 (range 2–30), and the average number of years of smoking was 11.6 (range 2–25) (Table S1).

General characteristics of the groups of GH and PE are presented in Table 1. The women in the GH group (compared to the normotensive women) were significantly older, had significantly higher pre-pregnancy BMI, gave birth significantly earlier, and gave birth to newborns with significantly lower weight. In the GH group, we found a statistically significantly higher number of smokers (ever before pregnancy) and smokers in the first trimester. Similar profiles of the results (but not identical) were found for comparison of normotensive controls and cases of PE (Table 1).

**Figure 1:**
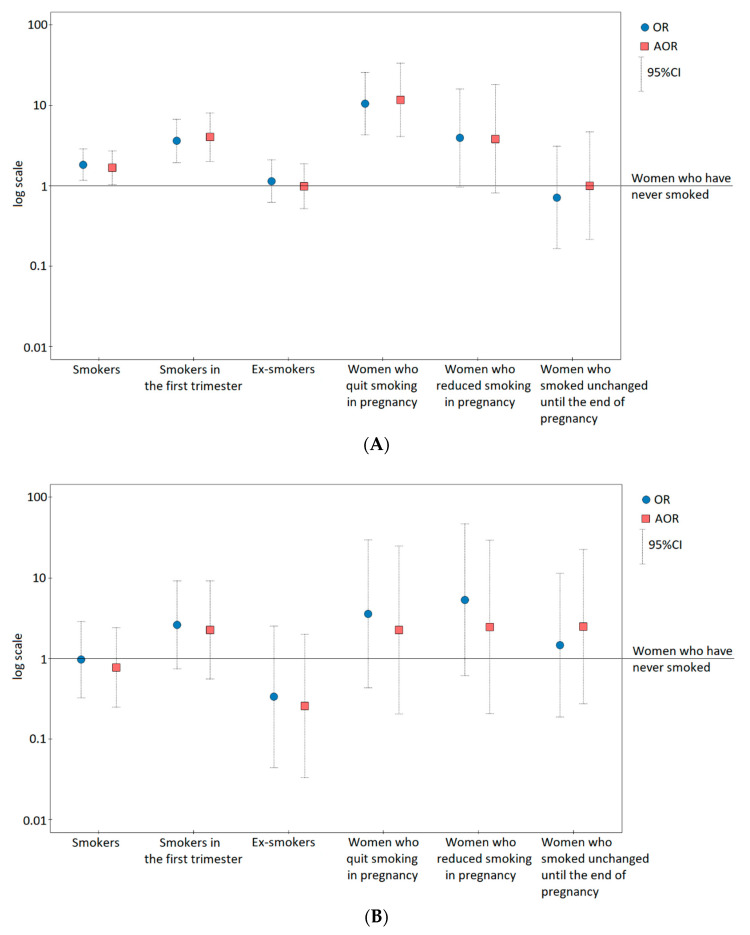
Odds ratios (and 95% confidence intervals) of gestational hypertension (**A**) and pre-eclampsia (**B**) for smoking categories on a logarithmic scale. The adjusted odds ratios (AOR) were calculated after adjusting for maternal age, primiparous women, pre-pregnancy body–mass index (BMI), and gestational weight gain outside the range of the recommendations regardless of the BMI category.

Figure 1 presents graphic pictures of the risk of GH (A) and PE (B) for studied smoking categories. Compared to the women who have never smoked, women who smoked in the first trimester as well as women who quit smoking during pregnancy and those who reduced smoking in pregnancy had a higher risk of GH and PE.

The AOR of both forms of hypertension for smoking categories are presented in Table 2, Table 3 and Table S2. Each analysis covered the cases and normotensive controls.

Compared to the women who have never smoked (Table 2), smoking ever before pregnancy was associated with a higher risk of GH (AOR = 1.68; *p* = 0.043), and with no effect on PE risk. Smokers in the first trimester had a 4.75-fold higher adjusted odds ratio of GH (*p* < 0.001) and a 2.60-fold higher odds ratio of PE (*p* = 0.136). Quitting smoking before pregnancy (ex-smokers) was associated with a lower odds ratio of GH and PE (OR/AOR < 1.0). However, quitting smoking during pregnancy was associated with a 11.63-fold higher adjusted risk of GH (*p* < 0.0001) and a 3.57-fold higher risk of PE (*p* = 0.238). Women who reduced smoking in pregnancy also had a higher risk of GH and PE, but in a statistically insignificant way.

The results for another reference category (smokers in the first trimester) are presented in Table S2.

Analyses of continuous variables (Table 3) showed that the number of years of smoking (ever before pregnancy) was a stronger risk factor of GH and PE than the number of cigarettes/day. An increase in the number of years of smoking of 1 year increases the adjusted risk of GH by 5% (AOR = 1.05, *p* = 0.006). The results for number of cigarettes/day and pack-years were statistically insignificant after adjustment. The profile of the results for PE risk was similar, but the results were statistically insignificant.

The odds ratios of PIH (and its forms) after dissection of the cohort into pre-pregnancy BMI categories are presented in Table 4 and Table S3.

Table 4 presents the results for all cases of PIH because some subgroups were small and / or included a small number of cases. The highest risk of PIH for smoking in the first trimester was found in women with pre-pregnancy underweight (OR = 22.00; *p* = 0.024).

Table S3 presents the results for GH and PE separately. The highest risk of these forms of hypertension for smoking in the first trimester was also found in women with pre-pregnancy underweight.

## 4. Discussion

In our prospective cohort study, we found that smoking in the first trimester increased the risk of isolated GH and PE, compared to the women who have never smoked. Smoking ever before pregnancy was associated with weaker results, and was associated with a higher risk of GH and with a negligible effect on the risk of PE. Compared to the women who have never smoked, quitting smoking before pregnancy (ex-smokers) was associated with a lower odds ratio of GH and PE. However, quitting smoking during pregnancy was associated with a higher risk of GH (much higher) and PE.

Our results are consistent with some reports in the literature [12,13,21,22,23]. In a population-based retrospective cohort study (USA) after excluding white-non-Hispanic women and Indian non-Hispanic women, Chang et al. found an increased risk of PE for smoking during pregnancy [21]. In a prospective cohort study of 508 single-pregnant women in the 16–22th week, Rauchfuss et al. found that women who never smoked had the lowest risk of pre-eclampsia; and the women who quit smoking during pregnancy increased the risk of PE [23]. In a population-based prospective cohort study, Bakker et al. found a slight increase in the risk of PE for smoking in the first trimester [22]. In a prospective study covering 605 women without chronic hypertension in the 24–26th week, Luo et al. found no effect of smoking during pregnancy on pre-eclampsia risk, but found an over 6-fold higher risk of PE for “previous smokers”, compared to “nonsmokers” [24].

Contrary to our results, many studies (and meta-analyses) showed a reduction in the risk of PE for smokers during pregnancy [10,12,13,25]. However, the strength of the relationship between smoking and PE (it is about 50% reduction in heavy smokers) is not as pronounced as with other smoking-related diseases [12]. 

The cited studies differed in their structure (retrospective or prospective studies) and methodology. The discrepancies found could be attributed to the risk differences of the surveyed populations, the different numbers of the studied groups, the different degree of matching of maternal features and various risk factors used to adjust the odds ratios.

The analysis of our results requires highlighting a few facts. The frequency of all PIH cases in our cohort (*n* = 137 = 15.0%) is 2–3 times higher than in other Polish studies [26,27]. The probable reason for this is the fact that the study was performed in a third-degree reference center, where women with risk factors report for additional tests. At the same time, women who developed pregnancy complications and those with risk factors (e.g., older or obese women) are more likely to cooperate. In our study, we found a small number of cases of pre-eclampsia (*n* = 24 = 2.6%), which is consistent with the latest FIGO report [1] in which the average occurrence of PE in the world was estimated at 2–5%. Importantly, in our study, we excluded a priori some risk factors, including multiple pregnancy, pre-existing hypertension, and other chronic diseases.

Secondly, in our study, 6.3% of women were smokers during pregnancy (in the first trimester), and 18.4% of participants were smokers ever before pregnancy. In addition, this is in line with the reports of recent meta-analyses presented in the Lancet; the prevalence of smoking during pregnancy was estimated to be 8.1% in the European Region (6.0% in Poland in 2015) [15]. These values are lower than presented in the World Health Organization (WHO) reports in previous years. In Poland, amendments to the Health Protection Act against the effects of tobacco and tobacco products took place in 2010 and 2012; in 2015 tobacco products were used by every fourth citizen (about every fifth woman) [14,15].

The mechanisms of the relationship between smoking and PIH are not clear. This is a heterogeneous disease entity. Isolated GH and PE may have common and different elements of pathophysiology. PE also has different phenotypes. In the pathomechanism of PE, the primary role of trophoblast invasion disorders in the period of placenta development and placental ischemia has been demonstrated. In addition, increased oxidative stress and inflammation as well as endothelial damage are main elements of the disease pathogenesis [2,5,6,7,28]. Importantly, different components of tobacco smoke may act on different processes or they may act differently on the same cellular processes, which may cause discrepancies between tests.

There are some mechanisms that may explain the increased risk of GH and PE in smokers [29]. Generally, pathomechanisms triggered by smoking (by the components of tobacco smoke) may affect the processes connected with the development of the placenta and cause damage to the structure and function of the vascular endothelium of the mother. It was shown that exposure to tobacco smoke triggers inflammation and oxidative stress in various organs and causes impairment of thrombotic events [17,29,30]. First, it has been previously shown that lower levels of micronutrients with antioxidant properties (e.g., selenium) may increase the risk of PIH [8,9,31,32]. At the same time, smoking has also been associated with lower selenium levels [8,9]. The increase in the level of lead and cadmium in smokers may cause a decrease in the concentration of antioxidant selenium that binds heavy metals [33]. Placental cadmium levels were also associated with increased risk of PE [34]. Secondly, it is possible that tobacco smoke affects the oxidative damage to endothelial cells (these cells synthesize endogenous nitric oxide NO, which has vasodilating properties). This can cause an imbalance between the action of vasoconstricting and vasodilating factors [35]. Thirdly, nicotine increases blood pressure by acting on the cardiovascular system [11]. However, it remains unexplained why smoking cessation during pregnancy increases the risk of hypertension. This requires testing in a large sample.

We consider our pilot study of the additive effect of smoking and the pre-pregnancy underweight (BMI < 18.5 kg/m^2^) on risk of hypertension in pregnancy to be extremely important (Table 4 and Table S3). It is possible that the result is associated with deficiencies of many nutrients in underweight people, including microelements and vitamins involved in oxidative balance, and inflammatory or immunological processes [36]. The sizes of the subgroups were small, but we believe that our result requires further research.

Some mechanisms may explain the reduction in the risk of PE in smokers [10]. For example, nicotine can have anti-inflammatory effects through the acetylcholine receptor subunit (α7-nAChR) and inhibition of pro-inflammatory cytokine production, which may attenuate hypertension caused by placental ischemia [11]. Exogenous carbon monoxide (CO) may have immunosuppressive effects and has the property of reducing vascular tone [11,12]. It was found that components of tobacco smoke reduce the dose-dependent level of sFlt-1 (an anti-angiogenic factor released by epithelial cells in the placenta), and previous studies correlated higher levels of sFlt-1 in early pregnancy with a higher risk of PIH/PE [10,37]. Nicotine inhibits the production of thromboxane A2, which may explain the results of a reduced risk of PE in smokers. Nicotine can reduce the volume of plasma by affecting the production of vaso-constrictive prostaglandins [10]. The possibility of permanent adaptation of the mother’s circulatory system is also taken into consideration in smokers [10]. 

### Limitations and Benefits

An advantage of this study was its prospective one-center cohort model. We have assessed the most possible smoking categories to get a full picture of the problem. We have taken into account many risk factors; some have been excluded a priori before the recruitment, and others have been used to correct the odds ratios. However, it is possible that there are other confounding variables that affect the results. The women came from one region, which matched them well in terms of the comparable level of prenatal care.

We are aware of the limitations of this study. We do not have any information on passive smoking. The participants reported their habits themselves. The subgroup sizes were smaller and met the minimum size requirements, but the limited group sizes account for a larger margin of error. The group of PE cases was not numerous.

## 5. Conclusions

In our study, the factors that increased the risk of isolated GH (statistically significant) and PE (statistically insignificant) were smoking in the first trimester and (paradoxically more strongly) smoking cessation during pregnancy. Our results were sustained after adjusting for many confounding variables.

Our results suggest that women of childbearing potential should be encouraged to quitting smoking before pregnancy. Efforts should be made to make women aware of the negative effects of smoking. Increased risk of hypertension in the women who quit smoking during pregnancy requires further research and clarification.

We also found that the risk of PIH for smoking in the first trimester increased more strongly in the women with pre-pregnancy underweight. Although this and some of our other results were obtained by examining small subgroups, further research in this topic is needed.

## Figures and Tables

**Table 1:** Maternal characteristics in the control and study groups.

	Normotensives (*n* = 775)	Cases (*n* = 137) *	
Maternal Characteristics	Mean (SD); Median or *n* (%)	Mean (SD); Median or *n* (%)	*p* **
	Controls (*n* = 775)	GH cases (*n* = 113)	
Maternal age (years)	33.5 (4.8); 35.0	35.0 (4.3); 36.0	0.005
Primiparous women	318 (41.0%)	53 (46.9%)	0.237
Prior PIH	4 (0.5%)	12 (10.6%)	<0.00001
Infertility treatment	29 (3.7%)	8 (7.1%)	0.097
Pre-pregnancy BMI (kg/m^2^)	23.3 (4.1); 22.5	26.7 (5.3); 25.5	< 0.001
GWG/week (kg/week) ***	0.35 (0.14); 0.34	0.38 (0.21); 0.38	0.057
Smokers	133 (17.2%)	31 (27.4%)	0.009
Smokers in the first trimester	37 (4.8%)	17 (15.0%)	0.00002
Fetal sex—son	405 (52.3%)	55 (48.7%)	0.476
Gestational age at delivery (week)	38.9 (1.6); 39.0	38.3 (2.2); 39.0	0.016
Newborn birthweight (g)	3416.5 (511.7); 3449.0	3174.1 (734.3); 3200.0	0.001
Gestational diabetes mellitus	121 (15.6%)	22 (19.5%)	0.298
	Controls (*n* = 775)	PE cases (*n* = 24)	
Maternal age (years)	33.5 (4.8); 35.0	34.1 (5.0); 35.0	0.434
Primiparous women	318 (41.0%)	12 (50.0%)	0.380
Prior PIH	4 (0.5%)	3 (12.5%)	<0.00001
Infertility treatment	29 (3.7%)	3 (12.5%)	0.031
Pre-pregnancy BMI (kg/m^2^)	23.3 (4.1); 22.5	26.5 (6.2); 25.0	0.008
GWG/week (kg/week) ***	0.35 (0.14); 0.34	0.42 (0.21); 0.38	0.155
Smokers	133 (17.2%)	4 (16.7%)	0.950
Smokers in the first trimester	37 (4.8%)	3 (12.5%)	0.084
Fetal sex—son	405 (52.3%)	13 (54.2%)	0.854
Gestational age at delivery (week)	38.9 (1.6); 39.0	35.1 (3.7); 36.0	< 0.001
Newborn birthweight (g)	3416.5 (511.7); 3449.0	2294.2 (927.5); 2445.0	< 0.001
Gestational diabetes mellitus	121 (15.6%)	3 (12.5%)	0.678
PE beginning: ≤31st week	-	7 (29.2%)	-
PE beginning 32–33rd week	-	4 (16.7%)	-
PE beginning ≥34th week	-	13 (54.2%)	-

* GH: gestational hypertension and PE: pre-eclampsia; ** The Mann–Whitney *U* test was used for comparisons of continuous variables; For binomial categories the Pearson chi-square test (or Fisher exact test when Cochran assumption was not met) was used (*p* < 0.05 was assumed to be significant); *** GWG: gestational weight gain.

**Table 2:** The adjusted odds ratios of gestational hypertension (GH) and pre-eclampsia (PE) for smoking categories.

Odds Ratios of Two Forms of GH and PE for Smoking Categories			
	Cases/Controls	OR (95% CI:); *p*	AOR * (95% CI:); *p*
Gestational hypertension (GH) risk			
Smokers **	31/133	1.83 (1.16–2.87); 0.009	1.68 (1.02–2.78); 0.043
Smokers in the first trimester	17/37	3.60 (1.94–6.68); <0.001	4.75 (2.34–9.65); <0.001
Ex-smokers	14/96	1.14 (0.62–2.09); 0.668	0.83 (0.41–1.66); 0.596
Women who have never smoked	82/642	1	1
Women who quit smoking in pregnancy ***	12/9	10.44 (4.27–25.53); <0.0001	11.63 (4.07–33.24); <0.0001
Women who reduced smoking in pregnancy	3/6	3.91 (0.96–15.95); 0.057	3.81 (0.81–18.05); 0.092
Women who smoked unchanged until the end of pregnancy	2/22	0.71 (0.16–3.08); 0.649	1.00 (0.21–4.64); 0.999
Women who have never smoked	82/642	1	1
Pre-eclampsia (PE) risk			
Smokers **	4/133	0.97 (0.33–2.87); 0.950	0.91 (0.29–2.88); 0.872
Smokers in the first trimester	3/37	2.60 (0.74–9.16); 0.136	2.51 (0.60–10.54); 0.208
Ex-smokers	1/96	0.33 (0.04–2.52); 0.288	0.31 (0.04–2.40); 0.260
Women who have never smoked	20/642	1	1
Women who quit smoking in pregnancy ***	1/9	3.57 (0.43–29.52); 0.238	2.25 (0.2–24.66); 0.508
Women who reduced smoking in pregnancy	1/6	5.35 (0.62–46.54); 0.129	2.47 (0.21–29.33); 0.474
Women who smoked unchanged until the end of pregnancy	1/22	1.46 (0.19–11.37); 0.718	2.49 (0.27–22.51); 0.418
Women who have never smoked	20/642	1	1

* AOR: adjusted odds ratios (CI—confidence intervals) calculated in the multivariate logistic regression (*p* < 0.05 was assumed to be significant); ** In these analyses, the adjusted odds ratios were calculated after adjusting for maternal age, primiparous, pre-pregnancy BMI, gestational weight gain outside the range of the recommendations regardless of the BMI category, prior PIH, and infertility treatment; *** In these analyses, the adjusted odds ratios were calculated after adjusting for maternal age, primiparous, pre-pregnancy BMI, gestational weight gain outside the range of the recommendations (the reason for the smaller number of confounders was the small number of cases in the subgroups).

**Table 3:** Adjusted odds ratios of gestational hypertension (GH) and pre-eclampsia (PE) for additional smoking categories (continuous variables).

Odds Ratios of PIH Forms for Continuous Variables		
	OR (95% CI:); *p*	AOR * (95% CI:); *p*
Gestational hypertension (GH) risk **		
For smokers (ever before pregnancy):		
Number of years of smoking (for 1 year)	1.06 (1.02–1.1); 0.006	1.05 (1.01–1.10); 0.031
Number of cigarettes per day (for 1 cigarette)	1.01(1.0–1.03); 0.148	1.01 (0.99–1.03); 0.299
Value of pack-years (for 1)	1.05 (1.0–1.10); 0.034	1.04 (0.99–1.09); 0.113
Pre-eclampsia (PE) risk ***		
For smokers (ever before pregnancy):		
Number of years of smoking (for 1 year)	1.05 (0.97–1.14); 0.256	1.04 (0.95–1.14); 0.352
Number of cigarettes per day (for 1 cigarette)	1.00 (0.96–1.05); 0.909	1.00 (0.95–1.04); 0.866
Value of pack-years (for 1)	1.02 (0.94–1.10); 0.616	1.01 (0.93–1.09); 0.891

* AOR: adjusted odds ratios (CI—confidence intervals) calculated in the multivariate logistic regression (*p* < 0.05 was assumed to be significant); ** In the analyses, the adjusted odds ratios were calculated after adjusting for maternal age, primiparous, pre-pregnancy BMI, gestational weight gain per week, prior PIH, and infertility treatment; *** In the analyses, the adjusted odds ratios were calculated after adjusting for maternal age, primiparous, pre-pregnancy BMI, gestational weight gain outside the range of the recommendations regardless of the BMI category (the reason for the smaller number of confounders was the small number of PE cases).

**Table 4:** The odds ratios of pregnancy-induced hypertension (PIH) for smoking in the first trimester, after dissection into pre-pregnancy BMI categories.

Odds Ratios of PIH for Smoking in the First Trimester, in the Pre-Pregnancy BMI Categories		
	Cases/Controls	PIH Risk OR * (95% CI:); *p*
Whole cohort		
Smoking in the first trimester	20/37	3.40 (1.90–6.09); <0.001
Women who have never smoked	102/642	1
Underweight		
Smoking in the first trimester	2/3	22.00 (1.52–319.48); 0.024
Women who have never smoked	1/33	1
Normal BMI		
Smoking in the first trimester	7/23	2.96 (1.21–7.26); 0.018
Women who have never smoked	47/457	1
Overweight		
Smoking in the first trimester	6/7	3.89 (1.2–12.63); 0.024
Women who have never smoked	24/109	1
Obesity		
Smoking in the first trimester	5/4	1.79 (0.44–7.23); 0.413
Women who have never smoked	30/43	1

* OR: crude odds ratios (CI—confidence intervals) calculated in the univariate logistic regression (*p* < 0.05 was assumed to be significant); PIH: pregnancy-induced hypertension; BMI: body–mass index.

## Supplementary Materials

The following are available online at https://www.mdpi.com/2077-0383/9/6/1743/s1, Table S1: Full characteristics of mothers in the control and PIH group; Table S2: The adjusted odds ratios of gestational hypertension (GH) and PE for all smoking categories; Table S3: The odds ratios of gestational hypertension (GH) and pre-eclampsia (PE) for smoking in the first trimester, after dissection into pre-pregnancy BMI categories.
