# Supplementary material for: The Influence of Various Smoking Categories on The Risk of Gestational Hypertension and Pre-Eclampsia
Source: J Clin Med. 2020 Jun 4;9(6):1743. doi: 10.3390/jcm9061743 (PMC7356904; doi:10.3390/jcm9061743)
Supplement: Supplementary file 1 [file jcm-09-01743-s001.pdf]

Supplementary Materials

Table S1. Full characteristics of mothers in the control and PIH group.

Maternal Characteristics	Normotensives (n = 775) * Mean (SD); Median or n (%)	PIH cases (n = 137) * Mean (SD); Median or n (%)	p **
**Basic**			
Maternal age (years)	33.5 (4.8); 35.0	34.8 (4.4); 35.0	0.005
Maternal age (range)	(18–45)	(19–45)	
Primiparous	318 (41.0%)	65 (47.5%)	0.161
Prior PIH	4 (0.5%)	15 (11.0%)	<0.00001
Infertility treatment	29 (3.7%)	11 (8.0%)	0.024
GWG/week (kg/week) ***	0.35 (0.14); 0.34	0.39 (0.21); 0.38	0.024
Education <12 years	48 (7.1%)	22 (18.3%)	0.0001
Pre-pregnancy BMI (kg/m²)	23.3 (4.1); 22.5	26.7 (5.4); 25.3	<0.001
Pre-pregnancy BMI (range)	(14.6–39.4)	(16.3–42.9)	
Pre-pregnancy underweight	44 (5.7%)	3 (2.2%)	0.089
**Smoking characteristics**			
Smokers	n = 133	n = 35	
How long (years) (available = 900)	1.30 (3.69); 0.0	2,39 (5.26); 0.0	0.011
No of cigarettes/day (available = 899)	1.68 (8.24); 0.0	2.94 (6.24); 0.0	0.005
Pack-years (available = 899)	0.75 (3.49); 0.0	1.61 (4.58); 0.0	0.008
Smokers in the first trimester	n = 37	n = 20	
How long (years) (available = 53)	11.1 (6.7); 10	12.6 (7.0); 13.5	0.497
Range	2–25	2–22	
Cigarettes/day (available = 54)	10 (5.7); 10	13.4 (7.1); 10	0.089
Range	2–20	3–30	
Pack-years (available = 53)	6.4 (6.5);3.8	9.2 (8.3); 5.6	0.156
Range	0.3–23.0	1.0–31.5	
Quit smoking during pregnancy	9 (24.3%)	13 (65.0%)	0.003
Reduced smoking during pregnancy	6 (16.2%)	4 (20.0%)	0.720
Smoked unchanged until the end of pregnancy	22 (59.5%)	3 (15.0%)	0.001
**Pregnancy results**			
Fetal sex- son	405 (52.3%)	68 (49.6%)	0.571
Gestational age at delivery (week)	38.9 (1.6); 39.0	37.7 (2.8); 38.0	<0.0001
Newborn birthweight (g)	3416.5 (511.7); 3449.0	3020.0 (838.0); 3130.0	<0.0001
Preeclampsia	–	24	–
Gestational hypertension	–	113	–
SABP after delivery (mmHg)	107.9 (10.8); 110.0	159.5 (18.2); 160.0	<0.001
DABP after delivery (mmHg)	66.8 (8.8); 70.0	100.9 (11.0); 100.0	<0.001
Gestational diabetes mellitus (GDM)	121 (15.6%)	25 (18.3%)	0.438

* Normotensive controls and cases of pregnancy-induced hypertension (PIH);

** The Mann-Whitney U test was used for comparisons of continuous variables; For binomial categories the Pearson chi-square test (or Fisher exact test when Cochran assumption was not met) was used (p <0.05 was assumed to be significant);

*** GWG: gestational weight gain; SABP—systolic arterial blood pressure; DABP—diastolic arterial blood pressure.

Table S2. The adjusted odds ratios of gestational hypertension (GH) and preeclampsia (PE) for all smoking categories.

	**Odds Ratios of Two Forms of GH and PE for All Smoking Categories**		
	**Cases/ controls**	**OR (95% CI:); *p***	**AOR * (95% CI:); *p***
**Gestational hypertension (GH) risk**			
Smokers	31/133	1.83 (1.16–2.87); 0.009	1.68 (1.02–2.78); 0.043
Smokers in the first trimester	17/37	3.60 (1.94–6.68); <0.001	4.75 (2.34–9.65); <0.001
Ex-smokers	14/96	1.14 (0.62–2.09); 0.668	0.83 (0.41–1.66); 0.596
Women who have never smoked **	82/642	1	1
Women who quit smoking in pregnancy	12/9	10.44 (4.27–25.53); <0.0001	11.63 (4.07–33.24); <0.0001
Women who reduced smoking in pregnancy	3/6	3.91 (0.96–15.95); 0.057	3.81 (0.81–18.05); 0.092
Women who smoked unchanged until the end of pregnancy	2/22	0.71 (0.16–3.08); 0.649	1.00 (0.21–4.64); 0.999
Women who have never smoked ***	82/642	1	1
Ex-smokers	14/96	0.32 (0.14–0.71); 0.005	0.14 (0.05–0.41); <0.001
Smokers in the first trimester **	17/37	1	1
Women who quit smoking in pregnancy	12/9	7.47 (2.07–27.0); 0.002	13.54 (2.26–81.1); 0.004
Women who reduced smoking in pregnancy	3/6	1.11 (0.24–5.08); 0.896	1.01 (0.17–5.90); 0.994
Women who smoked unchanged until the end of pregnancy	2/22	0.09 (0.02–0.46); 0.004	0.06 (0.01–0.42); 0.005
Smokers in the first trimester *** #	17/37	1	1
**Preeclampsia (PE) risk**			
Smokers	4/133	0.97 (0.33–2.87); 0.950	0.91 (0.29–2.88); 0.872
Smokers in the first trimester	3/37	2.60 (0.74–9.16); 0.136	2.51 (0.60–10.54); 0.208
Ex-smokers	1/96	0.33 (0.04–2.52); 0.288	0.31 (0.04–2.40); 0.260
Women who have never smoked **	20/642	1	1
Women who quit smoking in pregnancy	1/9	3.57 (0.43–29.52); 0.238	2.25 (0.2–24.66); 0.508
Women who reduced smoking in pregnancy	1/6	5.35 (0.62–46.54); 0.129	2.47 (0.21–29.33); 0.474
Women who smoked unchanged until the end of pregnancy	1/22	1.46 (0.19–11.37); 0.718	2.49 (0.27–22.51); 0.418
Women who have never smoked ***	20/642	1	1
Ex-smokers	1/96	0.13 (0.01–1.28); 0.08	0.21 (0.01–5.12); 0.335
Smokers in the first trimester **	3/37	1	1
Women who quit smoking in pregnancy	1/9	1.56 (0.13–19.24); 0.731	1.00 (0.01–80.05); 1.0
Women who reduced smoking in pregnancy	1/6	2.58 (0.20–33.24); 0.467	-
Women who smoked unchanged until the end of pregnancy	1/22	0.34 (0.03–4.11); 0.397	0.35 (0.01–20.21); 0.612
Smokers in the first trimester ***,#	3/37	1	1

* AOR: adjusted odds ratios (CI—confidence intervals) calculated in the multivariate logistic regression (*p* <0.05 was assumed to be significant);

** In the analyses, odds ratios were calculated after adjustment for maternal age, primiparous, pre-pregnancy BMI, gestational weight gain outside the range of the recommendations regardless of the BMI category, prior PIH and infertility treatment;

*** In the analyses, odds ratios were calculated after adjustment for maternal age, primiparous, pre-pregnancy BMI, gestational weight gain outside the range of the recommendations; # The reference category were variables other than those studied.

Table S3. The odds ratios of gestational hypertension (GH) and preeclampsia (PE) for smoking in the first trimester, after dissection into pre-pregnancy BMI categories.

	**Odds Ratios of GH and PE for Smoking in the First Trimester, in the Pre-Pregnancy BMI Categories**			
	**Cases/ Controls**	**GH risk OR * (95% CI:); *p***	**Cases/ Controls**	**PE risk OR * (95% CI:); *p***
**Whole cohort**				
Smoking in the first trimester	17/37	3.60 (1.94–6.68); <0.001	3/37	2.60 (0.74–9.16); 0.136
Women who have never smoked	82/642	1	20/642	1
**Underweight**				
Smoking in the first trimester	1/3	-	1/3	11.0 (0.54–223.89); 0.119
Women who have never smoked	0/33	1	1/33	1
**Normal BMI**				
Smoking in the first trimester	6/23	2.98 (1.15–7.74); 0.025	1/23	2.84 (0.34–24.05); 0.339
Women who have never smoked	40/457	1	7/457	1
**Overweight**				
Smoking in the first trimester	6/7	4.67 (1.42–15.36); 0.011	0/7	-
Women who have never smoked	20/109	1	4/109	1
**Obesity**				
Smoking in the first trimester	4/4	1.96 (0.45–8.57); 0.374	1/4	1.34 (0.13–13.64); 0.803
Women who have never smoked	22/43	1	8/43	1

* OR: crude odds ratios (CI—confidence intervals) calculated in the univariate logistic regression (p <0.05 was assumed to be significant); BMI: body mass index; GH: isolated gestational hypertension; PE: preeclampsia.
